# Presence of Extra-Criteria Antiphospholipid Antibodies Is an Independent Risk Factor for Ischemic Stroke

**DOI:** 10.3389/fcvm.2021.665741

**Published:** 2021-05-03

**Authors:** Laura Naranjo, Fernando Ostos, Francisco Javier Gil-Etayo, Jesús Hernández-Gallego, Óscar Cabrera-Marante, Daniel Enrique Pleguezuelo, Raquel Díaz-Simón, Mercedes Cerro, David Lora, Antonio Martínez-Salio, Antonio Serrano

**Affiliations:** ^1^Healthcare Research Institute, Hospital Universitario 12 de Octubre, Madrid, Spain; ^2^Immunology Department, Hospital Universitario 12 de Octubre, Madrid, Spain; ^3^Faculty of Medicine, Universidad Complutense de Madrid, Madrid, Spain; ^4^Neurology Department, Hospital Universitario 12 de Octubre, Madrid, Spain; ^5^Internal Medicine Department, Hospital Universitario 12 de Octubre, Madrid, Spain; ^6^Department of Nursing, Hospital Universitario 12 de Octubre, Madrid, Spain; ^7^Epidemiology Department, Healthcare Research Institute, Hospital Universitario 12 de Octubre, Madrid, Spain; ^8^Biomedical Research Centre Network for Epidemiology and Public Health (CIBERESP), Madrid, Spain; ^9^Faculty of Statistical Studies, Universidad Complutense de Madrid, Madrid, Spain

**Keywords:** antiphospholipid syndrome, ischemic stroke, antiphospholipid antibodies, IgA anti-b2-glycoprotein-I antibodies, thrombosis

## Abstract

**Background:** Ischemic stroke is the most common and severe arterial thrombotic event in Antiphospholipid syndrome (APS). APS is an autoimmune disease characterized by the presence of thrombosis and antiphospholipid antibodies (aPL), which provide a pro-coagulant state. The aPL included in the classification criteria are lupus anticoagulant, anti-cardiolipin (aCL) and anti-β2-glycoprotein-I antibodies (aB2GPI) of IgG and IgM isotypes. Extra-criteria aPL, especially IgA aB2GPI and IgG/IgM anti-phosphatidylserine/prothrombin antibodies (aPS/PT), have been strongly associated with thrombosis. However, their role in the general population suffering from stroke is unknown. We aim (1) to evaluate the aPL prevalence in ischemic stroke patients, (2) to determine the role of aPL as a risk factor for stroke, and (3) to create an easy-to-use tool to stratify the risk of ischemic stroke occurrence considering the presence of aPL and other risk factors.

**Materials and Methods:** A cohort of 245 consecutive ischemic stroke patients was evaluated in the first 24 h after the acute event for the presence of classic aPL, extra-criteria aPL (IgA aB2GPI, IgG, and IgM aPS/PT) and conventional cardiovascular risk factors. These patients were followed-up for 2-years. A group of 121 healthy volunteers of the same age range and representative of the general population was used as reference population. The study was approved by the Ethics Committee for Clinical Research (Reference numbers CEIC-14/354 and CEIC-18/182).

**Results:** The overall aPL prevalence in stroke patients was 28% and IgA aB2GPI were the most prevalent (20%). In the multivariant analysis, the presence of IgA aB2GPI (OR 2.40, 95% CI: 1.03–5.53), dyslipidemia (OR 1.70, 95% CI: 1.01–2.84), arterial hypertension (OR 1.82, 95% CI: 1.03–3.22), atrial fibrillation (OR 4.31, 95% CI: 1.90–9.78), and active smoking (OR 3.47, 95% CI: 1.72–6.99) were identified as independent risk factors for ischemic stroke. A risk stratification tool for stroke was created based on these factors (AUC: 0.75).

**Conclusions:** IgA aB2GPI are an important independent risk factor for ischemic stroke. Evaluation of aPL (including extra-criteria) in cardiovascular risk factor assessment for stroke can potentially increase the identification of patients at risk of thrombotic event, facilitating a decision on preventive treatments.

## Introduction

Stroke is a very serious life-threatening medical condition. It is the second leading cause of death and one of the most important causes of disability worldwide (50% of survivors will be permanently disabled) (1, 2). Strokes can be classified into two main types: ischemic strokes (about 90%) caused by an obstruction of blood supply, and haemorrhagic strokes due to the rupture of blood vessels (3). Ischemic stroke can be due to intracranial thrombosis or embolism, either from atherosclerotic plaque in the aortic arch, cervical arteries or the heart (4). When a cerebral artery is occluded by a clot and blood flow decreases, the oxygen and nutrients supply is interrupted and neuronal electrical function ceases. If circulation isn't restored quickly, diverse mechanisms of ischemic damage are involved in neuronal death and irreversible tissue injury causing long-term disability or even death (5).

Stroke is a complex disease with numerous aetiological factors and has been associated with numerous risk factors such as age, hypertension, diabetes mellitus, dyslipidemia, diet, smoking, or alcohol consumption (6). Furthermore, the presence of other previous diseases such as carotid artery disease, atrial fibrillation, mitral stenosis, coronary artery disease, and antiphospholipid syndrome (APS) are considered as facilitators of the appearance of arterial occlusions by thrombotic or cardioembolic mechanisms (7, 8). APS is a systemic autoimmune disease characterized by venous or arterial thrombotic events and/or gestational morbidity in patients positive for antiphospholipid antibodies (aPL). The presence of aPL contributes to the activation of immune cells, platelets, trophoblast, and endothelial cells (9–13) through the interaction of B2GPI-antibody complexes with several membrane receptors such as toll-like receptors (TLR2, TLR4), annexin 2 or LDL receptor-related protein 8 (LRP8) (14). Inflammatory mechanisms mediated by these complexes may be due to the release of neutrophil extracellular traps (NETs) and the complement activation. Activation of endothelial cells causes the loss of its anticoagulant properties and the acquisition of a pro-inflammatory and pro-coagulant phenotype (15). B2GPI-antibody complexes also reduce the tissue factor pathway inhibitor and activated protein C activity (16). However, although aPL are present, thrombotic events only occur occasionally, suggesting that aPL-associated clotting must be triggered by an additional “second hit” that involves a strong activation of the innate immunity, such as surgery, trauma, or infection (17). This “second hit” may induce the exposition of cryptic epitopes not previously accessible.

To date, there are no diagnostic criteria for APS. Classification criteria (clinical and laboratory criteria) were updated at a workshop in Sydney (Australia, 2004) in order to identify patients with APS for research purposes (18). The classification of a patient as APS requires the combination of one clinical and one laboratory criterion. Clinical criteria include arterial, venous or small vessel thrombosis in any tissue or organ, and/or pregnancy morbidity (19). The laboratory criteria are comprised by lupus anticoagulant (LA), anti-cardiolipin antibodies (aCL), and anti-β2-glycoprotein-I antibodies (aB2GPI), both of IgG or IgM isotypes. The importance of ischemic stroke in APS was already specified in the initial description of the syndrome in 1983 (20, 21) and the presence of aPL has been reported as an independent risk factor for the etiology of ischemic stroke (22–29).

There are patients who present APS clinical features where the evaluation of aPL recognized in the classification criteria is systematically negative. To describe these types of patients, Hughes and Khamashta defined the concept of “seronegative APS” and a growing interest in the development of new biomarkers that improve the diagnostic accuracy of APS has emerged (30). New aPL associated with APS clinical manifestations but not included in the current classification criteria have been described and are called extra-criteria aPL (31). Among them, aB2GPI of IgA isotype, IgG and IgM anti-phosphatidylserine/prothrombin (aPS/PT) antibodies, and IgG anti-domain I of B2GPI antibodies stand out for their strong association with APS clinical characteristics and for the existence of well-standardized diagnostic tests (32–34). Although IgA aB2GPI were not included in the APS classification criteria, their clinical importance has increased over the last 16 years (35–39). Since 2010, the task force of the 13th International Congress on Antiphospholipid Antibodies recommended testing for IgA aB2GPI in negative cases for IgG and IgM isotypes where APS is still suspected (40). The presence of IgA aB2GPI was demonstrated as a risk factor for the appearance of thrombosis and stroke. Asymptomatic IgA aB2GP1 carriers had a higher incidence of strokes than negative aPL people (OR 5.17, 95% CI: 1.13–23.59) within a 5-year follow-up study (37).

In this work, we analyse the prevalence of aPL (classic and extra-criteria) and well-known cardiovascular risk factors in a consecutive series of 245 patients with acute ischemic stroke and in a control group representing the general population. In addition, we propose a scoring model to stratify the risk of ischemic stroke considering the risk factors associated with this disorder (including aPL) that can be helpful to evaluate patients for preventive treatments.

## Materials and Methods

### Study Design

We performed a prospective and observational study that included 245 patients who suffered from acute ischemic stroke who were followed-up for 2 years. The presence of aPL was determined in the first 24 h after the acute event.

In this work, we aim (1) to analyse the prevalence of aPL (classic and extra-criteria) in ischemic stroke patients compared to a control population, and to assess the clinical evolution of aPL-positive patients in relation to those negative in a 2-year follow-up; (2) to quantify the role of aPL as a risk factor for ischemic stroke; and (3) to propose a risk stratification model based on the independent risk factors associated with stroke.

### Ischemic Stroke Patients

Two hundred and sixty-eight consecutive subjects with suspected acute stroke were attended at the Stroke Unit of the Hospital 12 de Octubre (Madrid, Spain) from November 1st 2017 to April 6th 2018. These 268 patients constitute the total number of patients who attended the Emergency Room for suspected stroke in this period. All patients were assessed by the on-duty neurologists at admission.

Patients underwent an emergency study including blood analysis (routine laboratory test, complete blood count, and coagulation test), electrocardiography study, cerebral imaging by computer tomography (CT), and CT-angiography. Complementary tests were performed depending on the patient's clinical condition and aetiological suspicion, including duplex ultrasound of intracranial, and supra-aortic vessels, transthoracic magnetic resonance imaging, transoesophageal echocardiography, or Holter ECG monitoring. Detailed information on vascular risk factors including hypertension, cardiac disease, hiperlipemia, diabetes mellitus, cigarette smoking, or alcohol intake was collected (defined below).

Twenty-three subjects were excluded due to a non-vascular origin of the event or a stroke not confirmed by brain imaging study. Therefore, 245 patients with a confirmed diagnosis were included (Supplementary Figure 1) and followed-up for 2 years. Stroke severity was assessed according to the National Institute of Health Stroke Scale (NIHSS) and the level of functional independence by the modified Rankin Score (mRS).

Patients whose symptoms were compatible with ischaemic stroke were treated with systemic intravenous thrombolysis using intravenous tissue plasminogen activator (IV-tPA) or by mechanical thrombectomy according to the Stroke Care Plan.

### Control Group and Reference Population

In order to compare the aPL prevalence between ischemic stroke patients and the healthy population, aPL were evaluated in a group of 501 anonymous blood donors (age, sex, and clinical data unknown). The blood donor group has the disadvantage that it does not include individuals over 65 years old, and those aged above 50 are underrepresented (age bias).

A second control group of 121 healthy individuals (reference population) representative of the general adult population of our area was constituted by 33 volunteers in the age range of 18–50 recruited consecutively at the blood bank's donor room and 88 volunteers aged over 50. Older participants were recruited from people who underwent a preoperative study for cataract surgery or other minor conditions (not related to any vascular pathology) and had no symptoms at the time of the medical examination except for minor age-related symptoms. This reference population and stroke patients were similar in both ethnicity (96% Caucasians) and area of residence. Individuals with antecedents of cardiovascular disease, neoplasia, or serious disease were excluded. Clinical and laboratory data of the 121 members of the reference population were stored in an anonymized database.

### Definitions

*Ischemic stroke:* was defined as rapidly developing signs of focal or global disturbance of cerebral functions, lasting more than 24 h or leading to death, with no apparent cause other than that of vascular origin. Stroke was confirmed by brain imaging study.

*Arterial hypertension:* was defined as systolic blood pressure >140 mmHg or diastolic blood pressure >90 mmHg recorded on different days during evaluations prior to the stroke (with evidence of at least 2 readings) or use of medication.

*Diabetes mellitus:* was defined as hyperglycemia resulting from defects of insulin secretion, insulin action, or both. It was diagnosed by medical evaluation.

*Dyslipidemia:* was defined as serum cholesterol concentrations >220 mg/dL, LDL >130mg/dL, triglycerides >150 mg/dL or use of medication in patients with dyslipidemia antecedents.

*Smoking:* was considered in active or former smokers.

*Atrial fibrillation:* was confirmed by electrocardiogram performed during evaluations prior to the stroke or at time of admission.

*Alcohol consumption*: was defined as >60 g/day in active or former drinkers.

Obesity: was considered when the body mass index (BMI) was >30, or in accordance with the current and past medical history.

*Ischaemic heart disease*: was considered when a history of angina or myocardial infarction was present.

*Peripheral artery disease:* was defined as medical diagnosis of intermittent claudication during exercise.

*Classic aPL:* any of the aPL included in the updated APS classification criteria excluding lupus anticoagulant.

*Extra-criteria aPL:* aPL not included in the updated APS classification criteria (IgA aβ2GPI and aPS/PT antibodies of IgG and IgM isotypes).

### Autoantibodies Determination

Both classic aPL and extra-criteria aPL levels were quantified in the first 24 h after the acute event at the Autoimmunity Laboratory of the Immunology Department. Classic aPL (aCL and aB2GPI of IgG and IgM isotypes) were quantified using BioPLex 2200 multiplex immunoassay system APLS (Bio-Rad, Hercules CA, USA). Antibody levels higher than 18 U/ml were considered positive for classic aPL (99th percentile of a healthy population, *N* = 270).

For extra-criteria aPL, IgA aB2GPI were quantified by enzyme-linked immunosorbent assay (ELISA) using the QUANTA Lite B2 GPI IgA (INOVA Diagnostics Inc., San Diego, CA, USA), and aPS/PT of IgG and IgM isotypes were evaluated using QUANTA Lite aPS/PT (INOVA DIAGNOSTICS, San Diego, CA, USA). The cut-off values considered positive were >20 U/ml for IgA anti-B2GPI, >30 U/ml for IgG aPS/PT, and >40 U/ml for IgM aPS/PT (41), based on the 99th percentile of a healthy population (*N* = 718). The donor population and the stroke cohort were similar in both ethnicity and area of residence.

### Ethical Issues

Individuals were included in the study in accordance with the Declaration of Helsinki. To assure the data anonymity, including both sera (blood drawn) and clinical data, a blind code was assigned to each patient. Approval was obtained from the Ethics Committee for Clinical Research of Hospital 12 de Octubre and a favorable report was received (Reference numbers CEIC-14/354 and CEIC-18/182). Written informed consents were obtained from all patients and the reference population.

### Statistical Methods

Demographic, clinical, and pathological characteristics of patients were described by the median with interquartile range (IQR) or relative frequencies. Comparisons of non-normally distributed scaled variables were performed using the Wilcoxon-Mann-Whitney test. The Kolmogorov-Smirnov test was used to evalaute the normality of a distribution.

Comparisons between qualitative variables were determined with Pearson's Chi-square test or Fisher's exact test, where appropriate. The relative measure of an effect was expressed as odds ratio (OR), indicating a 95% confidence interval (95% CI).

Logistic regression analysis was used to estimate the association between risk factors and the outcome (stroke) (42). The model discrimination was quantified using an area under a receiver operating characteristic (ROC) curve and the agreement between predicted and observed probabilities using the Hosmer-Lemeshow test. The intercept used to estimate the ischemic stroke risk was adjusted for the prevalence of ischemic stroke in the general population (43). A scoring system was created based on the regression coefficient. Different score values were evaluated in terms of sensitivity, specificity, likelihood of positive and negative ratio with their corresponding confidence intervals (44, 45).

Probabilities <0.05 were considered significant. Statistical analysis of data were performed using *MedCalc* Statistical Software version 19.5 (MedCalc Software, Ostend, Belgium).

## Results

### Patient's Characteristics and Conventional Cardiovascular Risk Factors

The stroke cohort had a similar proportion of men (131, 53.5%) and women (114, 46.5%). Men were significantly younger than women (*p* < 0.001), with a median age of 67 (IQR: 57–77) vs. 79 (IQR: 63–85), respectively. In the reference population, no significant age differences between men (median: 64 years, IQR: 50–75) and women (median: 70 years, IQR: 50–75) were detected (*p* = 0.963) (Table 1). Comparing both cohorts, women were significantly older in the stroke cohort (*p* < 0.001) but no significant differences in the male age were observed (*p* = 0.091).

**Table 1:** Clinical characteristics, conventional cardiovascular risk factors, and antiphospholipid antibodies in the ischemic stroke cohort and the reference population.

**Variables**	**Reference population (*****N*** **=** **121)**		**Stroke patients (*****N*** **=** **245)**						
	**N/median**	**%/IQR**	**N/median**	**%/IQR**	***p*-value**	**OR**	**95% CI**	**AUC**	**95% CI**
Age (years)	66	49.8–75.0	72	60.0–82.3	<0.001	1.03	1.02–1.05	0.63	0.58–0.68
Sex (men)	61	50.4	131	53.5	0.582				
Dyslipidemia	43	35.5	136	55.5	<0.001	2.26	1.44–3.55	0.6	0.55–0.65
Diabetes mellitus	17	14.0	74	30.2	<0.001	2.65	1.48–4.73	0.58	0.53–0.63
Former smoker	20	16.5	46	18.8	0.587				
Active smoker	14	11.6	48	19.6	0.054	1.86	0.98–3.53	0.54	0.49–0.59
Arterial hypertension	56	46.3	174	71.0	<0.001	2.84	1.81–4.47	0.62	0.57–0.67
Atrial fibrillation	8	6.6	71	29.0	<0.001	5.76	2.67–12.43	0.61	0.56–0.66
Obesity	24	19.8	37	15.1	0.254				
IgG aB2GPI	0	0.0	5	2.0	0.114				
IgM aB2GPI	3	2.5	10	4.1	0.436				
IgA aB2GPI	8	6.6	49	20.0	<0.001	3.53	1.61–7.72	0.57	0.51–0.62
IgG aCL	1	0.8	4	1.6	0.536				
IgM aCL	3	2.5	10	4.1	0.436				
IgG/IgM aPS-PT	5	4.1	19	7.8	0.188				
Classic aPL	3	2.5	15	6.1	0.130				
Any aPL	16	13.2	70	28.6	0.001	2.63	1.45–4.76	0.57	0.51–0.62

*aB2GPI, anti-β2-glycoprotein-I antibodies; aCL, anti-cardiolipin antibodies; aPL, antiphospholipid antibodies; aPS/PT, anti-phosphatidylserine/prothrombin antibodies; AUC, area under the ROC curve*.

Furthermore, significant differences were found in the main cardiovascular risk factors between the reference population and stroke patients (Table 1): dyslipidemia (35.5 vs. 55.5%, *p* < 0.001), diabetes mellitus (14.0 vs. 30.2%, *p* < 0.001), arterial hypertension (46.3 vs. 71.0%, *p* < 0.001), and atrial fibrillation (6.6 vs. 29.0%, *p* < 0.001), respectively. No differences in sex (50.4 vs. 53.5%, *p* = 0.582) or presence of autoimmune/inflammatory diseases (5.0 vs. 9.4%, *p* = 0.141) were found between both populations. Other analytical parameters of stroke patients at admission are described in Supplementary Table 1.

### Prevalence of Antiphospholipid Antibodies

The prevalence of classic aPL in ischemic stroke patients was 6.1%. The prevalence of each classic aPL individually was 2.0% for IgG aB2GPI, 4.1% for IgM aB2GPI, 1.6% for IgG aCL, and 4.1% for IgM aCL antibodies. The prevalence of extra-criteria aPL was 7.8% for IgG/IgM aPS/PT, and 20% for IgA aB2GPI (*n* = 49) (Table 1). Eighty-two percent of IgA aB2GPI-positive patients were isolated positive (negative for other aPL).

**Figure 1:**
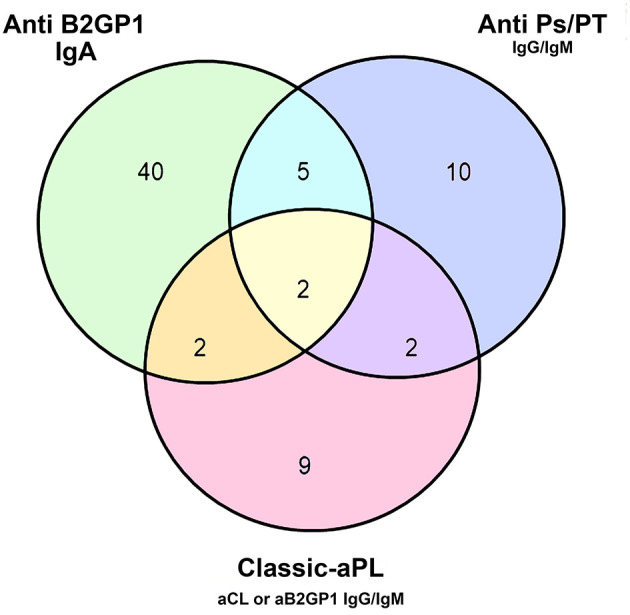
Number and type of aPL positivity in the ischemic stroke cohort. aB2GPI, anti-β2-glycoprotein-I antibodies; aCL, anti-cardiolipin antibodies; aPS/PT, anti-phosphatidylserine/prothrombin antibodies.

If we consider any aPL positivity (antibody levels above the cut-off for any classic or extra-criteria aPL), a total of 70 stroke patients (28.6%) were positive for any aPL and 40 of these were isolated positive for IgA aB2PI. The number and type of aPL positivity in stroke patients is described in Venn's diagrams in Figure 1.

Comparing the aPL prevalence between a control population of 501 blood donors and stroke patients, we found statistically significant differences in the prevalence of IgG aB2GPI (0.2 vs. 2.0%, *p* = 0.008), IgM aB2GPI (0.2 vs. 4.1%, *p* < 0.001), IgA aB2GPI (1.0 vs. 20.0%, *p* < 0.001), IgM aCL (0.0 vs. 4.1%, *p* < 0.001), and IgG/IgM aPS/PT (1.2 vs. 7.8%, *p* < 0.001), respectively (Supplementary Table 2). The absolute levels of criteria and extra-criteria aPL in both cohorts are represented in Supplementary Figure 2.

**Figure 2:**
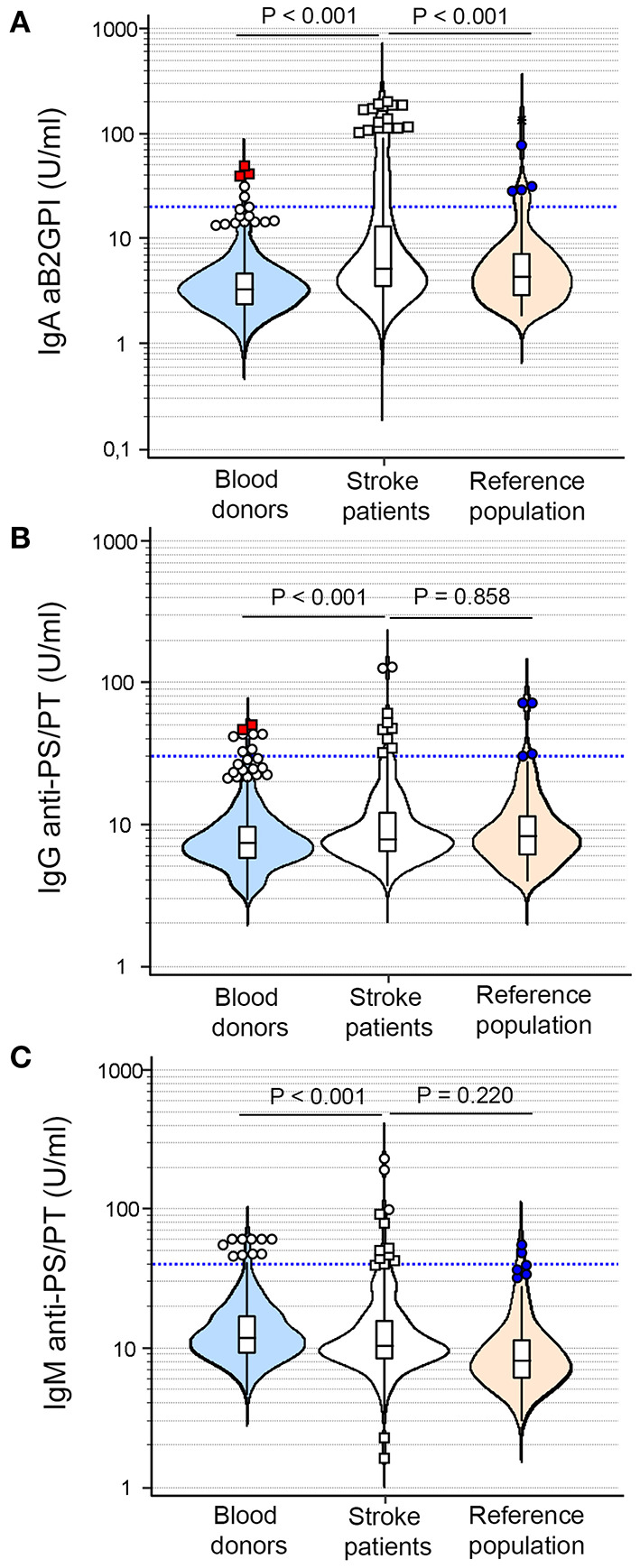
Distribution of extra-criteria aPL levels in blood donors (blue), stroke patients (white), and reference population (orange). **(A)** IgA anti-b2-glycoprotein-I antibodies. **(B)** IgG anti-phosphatidylserine/prothrombin antibodies. **(C)** IgM anti-phosphatidylserine/prothrombin antibodies. The cut-off of each antibody is represented by the blue lines.

However, using as control the reference population with a similar age range to stroke patients, no differences in the prevalence of most of these antibodies were found between both populations except for IgA aB2GPI, that was significantly higher in stroke patients (20%, *n* = 49) than in the reference population (6.6%, *n* = 8) (OR: 3.53, 95% CI: 1.61–7.72, *p* < 0.001). If we consider any aPL positivity, a total of 70 stroke patients (28.6%) vs. 16 individuals in the reference population (13.2%) were positive for any aPL (OR 2.63, 95% CI: 1.45–4.76, *p* = 0.001) (Table 1). Extra-criteria aPL levels in the reference population, blood donors, and stroke patients are represented in Figure 2. Mean and median levels of the different aPL are shown in Supplementary Table 3.

### Multivariate Analysis

The association of aPL and stroke-related conventional cardiovascular risk factors was examined by univariate analysis comparing stroke patients with the reference population. All factors with a *p*-value <0.10 in the univariate evaluation were included in a multivariate analysis model. The presence of any aPL (OR 2.28, 95% CI: 1.09–4.78, *p* = 0.028), dyslipidemia (OR 1.69, 95% CI: 1.01–2.83, *p* = 0.046), arterial hypertension (OR 1.82, 95% CI: 1.03–3.19, *p* = 0.038), atrial fibrillation (OR 4.40, 95% CI: 1.95–9.90, *p* < 0.001), and active smoker (OR 3.45, 95% CI: 1.71–6.93, *p* < 0.001) were independent and significant risk factors for ischemic stroke (Table 2A). A second multivariate model where the presence of any aPL was separated into classic aPL, IgG/IgM aPS/PT and IgA aB2GPI positivity was performed (Table 2B). This second model had a similar area under the ROC curve (0.756 in the first model vs. 0.758 in the second model) and the presence of IgA aB2GP1 was an independent risk factor for stroke, ranking in order of importance below atrial fibrillation, and smoking habit.

**Table 2:** **(A)** Multivariate analysis including variables with a *p*-value <0.10 identified in the univariate analysis, **(B)** Second multivariate analysis in which the presence of any positive aPL was separated into classic aPL, IgG/IgM aPS/PT, and IgA aB2GPI positivity, **(C)** Multivariate analysis including only the significant variables described in the second model.

**A) 1st Multivariate analysis**					
**Variable**	**Univariate**		**Multivariate**		
	**Odds ratio**	**95% CI**	**Odds ratio**	**95% CI**	***p*****-value**
Age (years)	1.03	1.02–1.05	1.01	0.99–1.03	0.213
Any positive aPL	2.63	1.45–4.76	2.28	1.09–4.78	**0.028**
Dyslipidemia	2.26	1.44–3.55	1.69	1.01–2.83	**0.046**
Diabetes mellitus	2.65	1.48–4.73	1.60	0.84–3.06	0.151
Arterial hypertension	2.84	1.81–4.47	1.82	1.03–3.19	**0.038**
Atrial fibrillation	5.76	2.67–12.43	4.40	1.95–9.90	** <0.001**
Active smoker	1.86	0.98–3.53	3.45	1.71–6.93	**0.001**
			**AUC 0.756**	**0.71–0.80**	
**B) 2nd Multivariate analysis**					
**Variable**	**Univariate**		**Multivariate**		
	**Odds ratio**	**95% CI**	**Odds ratio**	**95% CI**	***p*****-value**
Age (years)	1.03	1.02–1.05	1.01	0.99–1.03	0.218
Positive classic aPL	2.56	0.72–9.03	2.03	0.53–7.85	0.304
Positive IgA B2GPI	3.53	1.61–7.72	2.40	1.03–5.53	**0.042**
Positive IgG/IgM aPSPT	1.95	0.71–5.36	1.14	0.34–3.80	0.835
Dyslipidemia	2.26	1.44–3.55	1.70	1.01–2.84	**0.045**
Diabetes mellitus	2.65	1.48–4.73	1.59	0.83–3.05	0.161
Arterial hypertension	2.84	1.81–4.47	1.82	1.03–3.22	**0.041**
Atrial fibrillation	5.76	2.67–12.43	4.31	1.90–9.78	** <0.001**
Active smoker	1.86	0.98–3.53	3.47	1.72–6.99	** <0.001**
			**AUC 0.758**	**0.71–0.80**	
**C) Analysis of significant variables in second model**					
**Variable**	**Odds ratio**	**95% CI**	**Coefficient**	**Std. Error**	**Wald**
Positive IgA aB2GPI	2,60	1,13 to 5,94	0,954	0,422	5,106
Dyslipidemia	1,90	1,15 to 3,11	0,639	0,253	6,380
Arterial hypertension	2,26	1,36 to 3,75	0,814	0,259	9,877
Atrial fibrillation	5,01	2,26 to 11,10	1,611	0,406	15,724
Active smoker	3,23	1,63 to 6,41	1,172	0,35	11,207
Raw constant			−0,625	0,222	7,905
Adjusted^*^ constant			−4.810	0,222	7,905

*aB2GPI, anti-β2-glycoprotein-I antibodies; aCL, anti-cardiolipin antibodies; aPL, antiphospholipid antibodies; aPS/PT, anti-phosphatidylserine/prothrombin antibodies; AUC, area under the ROC curve*.

* *Adjusted to prevalence in general population. Significant values are written in bold*.

### Predictive Model Based on Independent Risk Factors

The exact probability of stroke can be determined using the logistic regression formula: probability = 1/[1 + exp –(intercept value + 0.8141^*^Arterial hypertension + 0.6392^*^Dyslipidemia + 1.6108^*^Atrial fibrillation + 1.1722^*^Active smoker + 0.9541^*^IgA aB2GPI Positive)]. The intercept value (−4.81) was adjusted for the prevalence of ischemic stroke in the general population by subtracting −4.18 (odds(3%)/odds(66.93%)) from the previous intercept (−0.62).

An easy risk-scoring system for stroke was constructed based on the independent variables identified in the multivariate model 2 (Table 2C). The points associated with each variable of the risk model and the probability of stroke according to the total score are described in Table 3. The total score can be calculated using this formula:

$$\begin{array}{l} Score=\left(Dyslipidemia\times 1\right)+\left(Hypertension\times 1\right)\\ +\left(IgAaB2GPI\times 2\right)+\left(Atrialfribrillation\times 3\right)\\ +\left(Activesmoking\times 2\right) \end{array}$$

For each patient, the value for the presence of atrial fibrillation, active smoking, IgA aB2GP1, dyslipidemia, and hypertension is 1 in this formula. The value for the absence of these conditions is 0. The ROC analysis of the risk predictive model presents an area under the curve of 0.75 (95% CI: 0.70–0.79). Positive and negative predictive values, diagnostic odds, and Youden index are described in Supplementary Table 4.

**Table 3:** Stroke risk scoring system.

**A) Score points associated with each variable**		
**Variable**	**Condition**	**Score**
Arterial hypertension	Absence	0 points
	presence	1 points
Dyslipidemia	Absence	0 points
	Presence	1 points
Atrial fibrillation	Absence	0 points
	Presence	3 points
Active smoker	Absence	0 points
	Presence	2 points
IgA anti-B2GPI Positive	Absence	0 points
	Presence	2 points
**B) Total score and stroke probability**		
**Total score**	**Stroke probability**	
0	0.008	
1	0.015	
2	0.029	
3	0.053	
4	0.095	
5	0.167	
6	0.275	
7	0.418	
8	0.576	
9	0.720	

***(A)***Points associated with each variable of the risk model. ***(B)***Probability of the stroke occurrence according to the total score. The score can be calculated using the formula: Score = (Dyslipidemia*1) + (Hypertension*1) + (IgA aB2GPI*2) + (Atrial fibrillation*3) + (Active smoking*2). The value for the presence and absence of the variables is 1 and 0, respectively.

**Figure 3:**
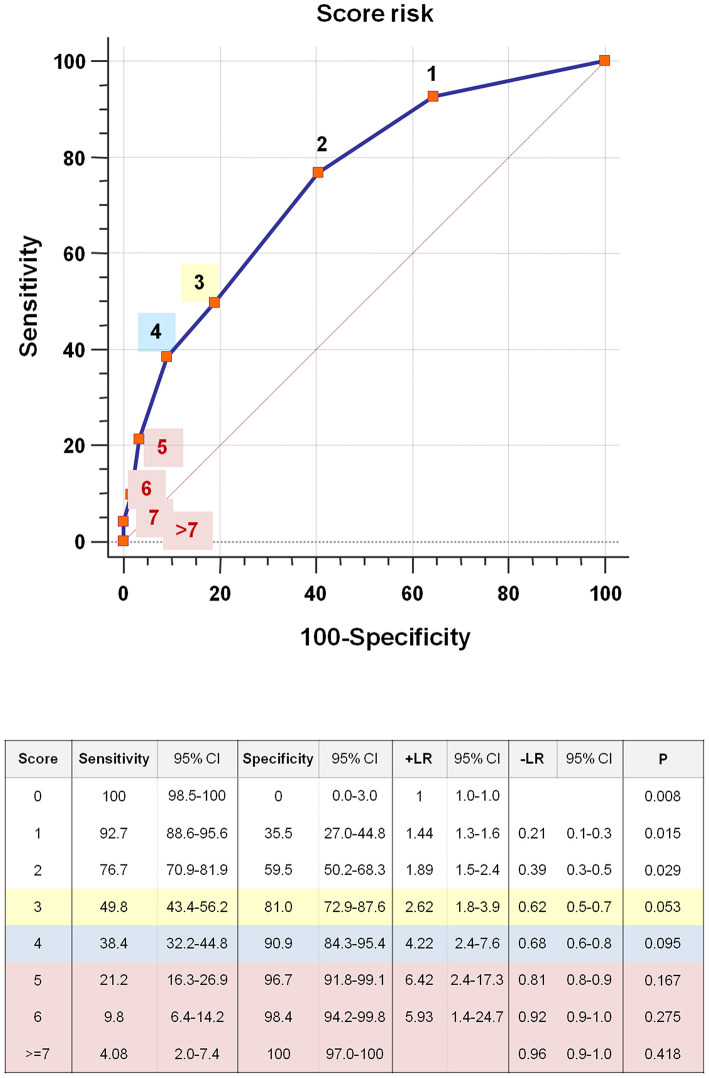
Risk stratification model, predicted probability of stroke, and representation of the score values.

Using the specificity score value, 3-risk categories were created (Figure 3): (1) moderate, specificity <80–90% (score = 3, yellow); (2) high, specificity of 90–95% (score = 4, blue), and (3) very high, specificity >95% (score ≥ 5, red). For example, if we had a patient with hypertension, positive for IgA aB2GPI, and smoker, the calculated score would be the following: Score = 0 + 1 + 2 + 0 + 2 = 5. The stroke probability associated with this score value would be 16.7% (Table 3).

### Clinical Evolution After a 2-Year Follow-Up

Differences in the level of functional independence using mRS between patients with any aPL and without aPL were not detected (OR 1.01, 95% CI: 0.87–1.15, *p* = 0.972). Besides, no significant differences between both populations were observed in the appearance of new strokes (OR 1.43, 95% CI: 0.48–4.32, *p* = 0.517), ischaemic heart disease (OR 0.81, 95% CI: 0.33–1.97, *p* = 0.638), peripheral artery disease (OR 1.39, 95% CI: 0.41–4.71, *p* = 0.590) or death (OR 0.99, 95% CI: 0.44–2.24, *p* = 0.980). Similarly, the presence of IgA aB2GP1 was not associated with the clinical prognosis, recurrence of vascular events or mortality.

## Discussion

The role of IgA aB2GPI in stroke etiology has been largely unstudied and its involvement in this pathology is unknown. In this work we demonstrate that IgA aB2GPI are the most prevalent autoantibodies in ischemic stroke patients and their presence constitutes an important independent risk factor associated with the occurrence of sporadic ischemic strokes, regardless of whether or not patients have a previous history of systemic autoimmune disease. The importance of IgA aB2GPI as a risk factor for stroke is higher than some conventional risk factors such as dyslipidemia or hypertension. Only smoking habit and presence of atrial fibrillation constitute risk factors of greater intensity than IgA aB2GPI.

Although aB2GPI of IgA isotype were not included in the APS classification criteria, their clinical importance has increased over the last 16 years (35–39) and in the 13th International Congress on Antiphospholipid Antibodies the task force recommended testing for IgA anti-B2GPI when other aPL are negative and APS is still suspected (40).

In clinical studies with APS and other autoimmune disease patients, control populations are usually blood donors, a very homogeneous group of people with good health, and an age range of 18–65 (mainly between 30 and 50 years). However, it has been known for a long time that stroke has a higher incidence in the elderly and the presence of autoantibodies (anti-nuclear, aPL, and anti-thyroid antibodies) is also more frequent in older populations than in young individuals (46, 47). The autoantibodies in the elderly can be pathology-related or age-related. Most of the age-related autoantibodies are scavenger antibodies (not associated with an autoimmune response) produced in response to an increase of apoptotic cells caused by tissue damage in the context of the senescence process (48). In order to minimize the influence of age as a confounding variable and to properly assess whether the presence of aPL is associated with a higher incidence of stroke, we used the reference population as control group of the healthy population with an age range similar to that of stroke patients.

Neurological involvement in APS is relatively frequent (49). It has long been known that ischemic stroke is the most common and severe arterial thrombotic event in APS, with significant morbidity (50, 51). There are several studies that reported an increased frequency of stroke in APS patients (52–54), with a prevalence of 19.8% for stroke in the Euro-Phospholipid Project (55). When this cohort was followed-up for 10 years, stroke was the most common thrombotic event that appeared in that period. Similarly, a well-known association of aPL and ischemic stroke has been described (56–61), highlighting a meta-analysis by Chighizola et al. (58) that describes an aPL prevalence of 10% in stroke patients and a study by Gaspersic et al. (29) that shows an aPL prevalence of 22% in stroke patients (including extra-criteria aPL), concluding that aPL represent an independent risk factor for cerebrovascular events. The lower prevalence of classic aPL in our group (6.1%) may be due to differences in the cut-off points and because we did not evaluate the lupus anticoagulant. Furthermore, an overall aPL frequency of 17.2% was reported in young patients (<50 years) with a stroke, supporting that aPL could be considered a leading cause of strokes below the age of 50 (62).

Despite the widespread association of criteria aPL with stroke, the role of extra-criteria aPL in ischemic stroke is not sufficiently known. In a previous study, our group observed a high incidence of thrombotic events (especially ischemic strokes) in asymptomatic carriers of isolated IgA aB2GPI, proposing a score to calculate the risk of thrombotic events (37). Besides, other published studies have also suggested a relevant role for this antibody in the stroke etiology (63, 64). In addition, the presence of criteria aPL has been reported as an independent risk factor for strokes (22–29).

The findings observed at initial evaluation of stroke patients were not related with the clinical evolution in the 2-year follow-up in terms of level of functional independence, recurrence of vascular events or mortality, suggesting that aPL have their role at the time of the event, and there are no differences from strokes caused by other reasons. Some previous studies have not found (60, 65) or have found a weak correlation (66) between serum aPL levels and the stroke severity or outcome.

Atrial fibrillation is a leading cause of stroke in the elderly and its prevalence increases with age (67). About a quarter of acute strokes in older patients are caused by atrial fibrillation, however, anticoagulation reduces the stroke risk by 70% in atrial fibrillation (68, 69). In this cohort, we found a prevalence of 29.0% for atrial fibrillation, which is in accordance with the prevalence described in other large cohorts of stroke patients [23.3% for patients aged between 64 and 74, 34.3% aged between 75 and 84 in the Barcelona Stroke Registry (7) or 20.1% in the stroke cohort of the Framingham Study (70)].

Up to 85% of all strokes could be prevented by acting on risk factors with effective medical interventions and modifying the lifestyle of patients (69). In patients with high thromboembolic risk, such as those with atrial fibrillation, the accumulated evidence advises the establishment of antithrombotic prophylaxis (71, 72). An effective thromboprophylaxis requires a balance between efficacy and safety: the benefits of prevention must outweigh the risk of bleeding, so tools which help decide when to start antithrombotic prophylaxis are very useful in clinical practice. The CHA_2_DS_2_-VASc score allows the estimation of stroke risk in the case of patients with atrial fibrillation based on the presence of different factors and assessing which should have anticoagulant treatment (73).

The inclusion of aPL testing in the profile of tests used in Vascular/Heart health screening could identify people at risk of thrombotic event which could be controlled with preventive treatments (74–76). However, when a patient presents IgA aB2GPI it is not clear whether the beneficial effects of an antithrombotic prophylaxis would justify assuming the inherent risks of the treatment. In this work, we propose a risk stratification model that allows the calculation of the risk of stroke taking into account the simultaneous presence of several risk factors, so it can be useful in helping decide on the convenience of establishing prophylactic treatments. In our model, we have observed that the presence of IgA aB2GPI as the only risk factor means a lower risk than atrial fibrillation. However, IgA aB2GPI-carriers with additional risk factors may have a higher risk than those with only atrial fibrillation. In this sense, the cumulative risk due to the presence of hypertension and dyslipidemia in an IgA aB2GPI-carrier would be greater than the risk of a patient with atrial fibrillation. The value of our prediction model should be verified by independent studies on other cohorts of patients.

### Limitations

This work has several limitations. Although, the sample size is large, it is a single center study and the findings described here should be evaluated in future multicentre studies. Another limitation is that we have no data about lupus anticoagulant in most patients of the study, since it is not routinely evaluated in patients with a first thrombotic event in our hospital (only in patients with recurrent thrombosis). However, this is partially compensated by the fact that patients were tested for anti-PS/PT. A study of Meroni's group showed that the good correlation between the levels of aPS/PT and LA supports the use of these antibodies as a surrogate test for LA (77). Subsequent studies also support that aPS/PT represent an additional diagnostic tool when LA test is not available or the results are uncertain (78, 79). The lack of a second aPL evaluation is another drawback of this study, but the systematic evaluation of IgA B2GPI in patients with APS has demonstrated that IgA aB2GPI positivity is extremely stable and persists for years. Six months after the first diagnosis, 96% of positive patients continue to be positive (80). The remaining cases were patients with antibody levels very close to the cut-off (gray zone) who became intermittently positive in successive samples. A similar situation occurs with aPL of IgG isotype. Besides, we cannot rule out the possible contribution of other unmeasured factors.

### Future Directions

Cerebrovascular events are one of the main causes of mortality with important social consequences but limited data exist about the frequency of non-conventional risk factors in this pathology. The potential role of IgA aB2GPI in stroke should be confirmed by further studies using immunoassays with appropriate and proven sensitivities.

In conclusion, this study demonstrates that IgA aB2GPI are an important independent risk factor for ischemic stroke. The incorporation of aPL evaluation (including extra-criteria aPL) in the assessment of cardiovascular risk factors for ischemic stroke during health examinations could increase the identification of patients at risk of thrombotic events, allowing evaluation to establish preventive treatments.

## Data Availability Statement

The raw data supporting the conclusions of this article will be made available by the authors, without undue reservation.

## Ethics Statement

The studies involving human participants were reviewed and approved by Ethics Committee for Clinical Research of Hospital 12 de Octubre (Reference numbers CEIC-14/354 and CEIC-18/182). The patients/participants provided their written informed consent to participate in this study.

## Author Contributions

AM-S and AS conceived and designed the study. FO, AM-S, JH-G, RD-S, and MC participated in the recruitment and screening of patients and in clinical data acquisition. LN, ÓC-M, DP, and FG-E performed laboratory tests and contributed to the collection of samples. LN, AS, and AM-S incorporated clinical and analytical information to the database, performed the first data analysis, and wrote the first draft of the article. AS and DL made the statistical analysis. LN and AS wrote the final draft of the article and made all the changes suggested by the co-authors. All authors contributed to the article and approved the submitted version.

## Conflict of Interest

The authors declare that the research was conducted in the absence of any commercial or financial relationships that could be construed as a potential conflict of interest.
