# Supplementary material for: Presence of Extra-Criteria Antiphospholipid Antibodies Is an Independent Risk Factor for Ischemic Stroke
Source: Front Cardiovasc Med. 2021 May 3;8:665741. doi: 10.3389/fcvm.2021.665741 (PMC8126615; doi:10.3389/fcvm.2021.665741)
Supplement: Supplementary file 2 [file Data_Sheet_1.docx]

Supplementary Material

**Supplementary Table S1.** Additional analytical parameters in stroke patients at hospital admission.

SD: standard deviation; IQR: interquartile range.

| **Variable** | **Mean** | **SD** | **Median** | **IQR** |
| --- | --- | --- | --- | --- |
| Hemoglobin (g/dL) | 13.4 | 1.9 | 13.6 | 11.9-14.6 |
| Erythrocytes (10^6^/mL) | 4.5 | 0.6 | 4.58 | 4.1-5 |
| Platelets (10^3^/mL) | 224.5 | 81.4 | 216 | 164.7-260 |
| Leukocytes (10^3^/mL) | 9.1 | 3.4 | 8.4 | 6.9-10.6 |
| LDH (IU/mL) | 258.6 | 67.5 | 256 | 220.7-299.5 |
| Ferritin (ng/mL) | 177.3 | 173.8 | 128.1 | 76.8-208.4 |
| C-reactive protein (mg/dL) | 1.4 | 2.3 | 0.51 | 0.2-1.2 |
| Total cholesterol (mg/dL) | 168.8 | 42.2 | 165 | 138.8-195.8 |
| Non-HDL Cholesterol (mg/dL) | 121.5 | 40 | 113.2 | 92.9-143.8 |
| Alanine aminotransferase (IU/mL) | 18 | 11.8 | 15.4 | 11-21.9 |
| Aspartate aminotransferase (IU/mL) | 21.7 | 10.5 | 19 | 15.5-25 |
| Creatinine (mg/dL) | 0.93 | 0.46 | 0.83 | 0.7-1 |

**Supplementary Table S2.** Prevalence of antiphospholipid antibodies in the control group of blood donors and ischemic stroke patients.

aB2GPI: anti-β2-glycoprotein-I antibodies; aCL: anti-cardiolipin antibodies; aPL: antiphospholipid antibodies; aPS/PT: anti-phosphatidylserine/prothrombin antibodies.

| **Antibodies** | **BLOOD DONORS**  (N=501) | | **STROKE PATIENTS**  (N=245) | | **p-value** |
| --- | --- | --- | --- | --- | --- |
|  | Number of positive | % | Number of positive | % |  |
| IgG aB2GPI | 1 | 0.2 | 5 | 2.0 | 0.008 |
| IgM aB2GPI | 1 | 0.2 | 10 | 4.1 | <0.001 |
| IgA aB2GPI | 5 | 1.0 | 49 | 20.0 | <0.001 |
| IgG aCL | 2 | 0.4 | 4 | 1.6 | 0.076 |
| IgM aCL | 0 | 0.0 | 10 | 4.1 | <0.001 |
| IgG/IgM aPSPT | 6 | 1.2 | 19 | 7.8 | <0.001 |
| Classic aPL  (IgG/IgM aCL/aB2GPI) | 3 | 0.6 | 15 | 6.1 | <0.001 |
| Any aPL | 39 | 7.8 | 70 | 28.6 | <0.001 |

**Supplementary Table S3**. Mean and median levels of antiphospholipid antibodies in the reference population, blood donors and stroke patients.

aB2GPI: anti-β2-glycoprotein-I antibodies; aCL: anti-cardiolipin antibodies; aPS/PT: anti-phosphatidylserine/prothrombin antibodies; SD: standard deviation; IQR: interquartile range.

| **Antibodies** | **BLOOD DONORS**  (n=501) | | | | | | **REFERENCE POPULATION**  (n=121) | | | | | | **STROKE PATIENTS**  (n=245) | | | | | |  |
| --- | --- | --- | --- | --- | --- | --- | --- | --- | --- | --- | --- | --- | --- | --- | --- | --- | --- | --- | --- |
|  | Mean | SD | Median | | | IQR | Mean | SD | Median | IQR | | | Mean | | SD | Median | | IQR |  |
| IgA aB2GP1 | 4,3 | 4,36 | 3,3 | 2,4-4,7 | | | 7,8 | 14,51 | 4,3 | 2,9-7,1 | | | 19,9 | | 37,63 | 5,1 | | 3,5-12,9 |  |
| IgG aB2GP1 | 1,6 | 1,66 | 1,4 | 1,4-1,4 | | | 1,8 | 1,73 | 1,4 | 1,4-1,4 | | | 3,4 | | 15,72 | 1,4 | | 1,4-1,4 |  |
| IgM aB2GP1 | 1,6 | 2,03 | 1,0 | 0,4-1,9 | | | 3,5 | 7,14 | 1,6 | 0,7-3,8 | | | 4,7 | | 15,28 | 1,2 | | 0,5-3,3 |  |
| IgG aCL | 1,9 | 1,80 | 1,6 | 1,6-1,6 | | | 2,3 | 2,31 | 1,6 | 1,6-1,6 | | | 3,6 | | 15,31 | 1,6 | | 1,6-1,6 |  |
| IgM aCL | 1,3 | 1,57 | 0,9 | 0,3-1,9 | | | 4,0 | 7,54 | 1,9 | 0,7-4,7 | | | 4,5 | | 15,22 | 1,1 | | 0,5-3,0 |  |
| IgG aPS/PT | 8,8 | 5,89 | 7,34 | 5,7-9,6 | | | 10,7 | 9,70 | 8,2 | 6,1-11,2 | | | 11,6 | | 13,16 | 7,9 | | 6,4-12,1 |  |
| IgM aPS/PT | 14,2 | 8,49 | 11,8 | 9,2-16,8 | | | 10,6 | 8,55 | 8,0 | 6,1-11,3 | | | 16,0 | | 21,46 | 10,4 | | 8,5-15,9 |  |
|  |  |  |  | |  | | | | | |  |  | |  | | |  | |  |

**Supplementary Table S4.** Score values and characteristics of the ROC curve. A) Score values with sensitivity, specificity and predictive values. B) Youden index.

+LR: positive likelihood ratio; -LR: negative likelihood ratio; PPV: positive predictive value; NPV: negative predictive value; CI: confidence interval.

| **A) Score values and statistical characteristics of the ROC curve** | | | | | | | | | | | | |  |  |
| --- | --- | --- | --- | --- | --- | --- | --- | --- | --- | --- | --- | --- | --- | --- |
| Score | Sensitivity | 95% CI | Specificity | | 95% CI | | +LR | 95% CI | | -LR | 95% CI | | PPV | NPV |
| 0 | 100 | 98.5 - 100 | 0.00 | | 0.0 - 3.0 | | 1.00 | 1 - 1 | |  |  | | 66.9 |  |
| 1 | 92.65 | 88.6 - 95.6 | 35.54 | | 27.0 - 44.8 | | 1.44 | 1.3 - 1.6 | | 0.21 | 0.1 - 0.3 | | 74.4 | 70.5 |
| **2** | 76.73 | 70.9 - 81.9 | 59.50 | | 50.2 - 68.3 | | 1.89 | 1.5 - 2.4 | | 0.39 | 0.3 - 0.5 | | 79.3 | 55.8 |
| 3 | 49.80 | 43.4 - 56.2 | 80.99 | | 72.9 - 87.6 | | 2.62 | 1.8 - 3.9 | | 0.62 | 0.5 - 0.7 | | 84.1 | 44.3 |
| 4 | 38.37 | 32.2 - 44.8 | 90.91 | | 84.3 - 95.4 | | 4.22 | 2.4 - 7.6 | | 0.68 | 0.6 - 0.8 | | 89.5 | 42.1 |
| 5 | 21.22 | 16.3 - 26.9 | 96.69 | | 91.8 - 99.1 | | 6.42 | 2.4 - 17.3 | | 0.81 | 0.8 - 0.9 | | 92.9 | 37.7 |
| 6 | 9.80 | 6.4 - 14.2 | 98.35 | | 94.2 - 99.8 | | 5.93 | 1.4 - 24.7 | | 0.92 | 0.9 - 1 | | 92.3 | 35.0 |
| 7 | 4.08 | 2.0 - 7.4 | 100 | | 97.0 - 100 | |  |  | | 0.96 | 0.9 - 1 | | 100.0 | 34.0 |
| >7 | 0.00 | 0- 1.5 | 100 | | 97.0 - 100 | |  |  | | 1 | 1 - 1 | |  | 33.1 |
| **B) Youden Index** | | | | | | | | | | | | | | |
| Statistic | | | | Value | | 95% CI | | | Associated  criterion | | | 95% CI | | |
| Area under the ROC curve | | | | 0.746 | | 0.698 - 0.790 | | |  | | |  | | |
| Youden index J | | | | 0.362 | | 0.264 - 0.461 | | | 2 | | | 1 - 3 | | |

**Supplementary Figure S1.** Study disposition. The cohort included 245 patients from the Stroke Unit with a confirmed ischemic stroke who were followed-up for 2 years.

aB2GPI: anti-β2-glycoprotein-I antibodies; aCL: anti-cardiolipin antibodies; aPS/PT: anti-phosphatidylserine/prothrombin antibodies.

**Supplementary Figure S2.** Distribution of aPL levels in blood donors (grey) and stroke patients (black). A) Classification criteria aPL. B) Extra-criteria aPL.

aB2GPI: anti-β2-glycoprotein-I antibodies; aCL: anti-cardiolipin antibodies; aPS/PT: anti-phosphatidylserine/prothrombin antibodies.

**Excel file**. Data on age, sex, presence of risk factors and score value for stroke patients.
