# Supplementary figures and images for: Presence of Extra-Criteria Antiphospholipid Antibodies Is an Independent Risk Factor for Ischemic Stroke
Source: Front Cardiovasc Med. 2021 May 3;8:665741. doi: 10.3389/fcvm.2021.665741 (PMC8126615; doi:10.3389/fcvm.2021.665741)

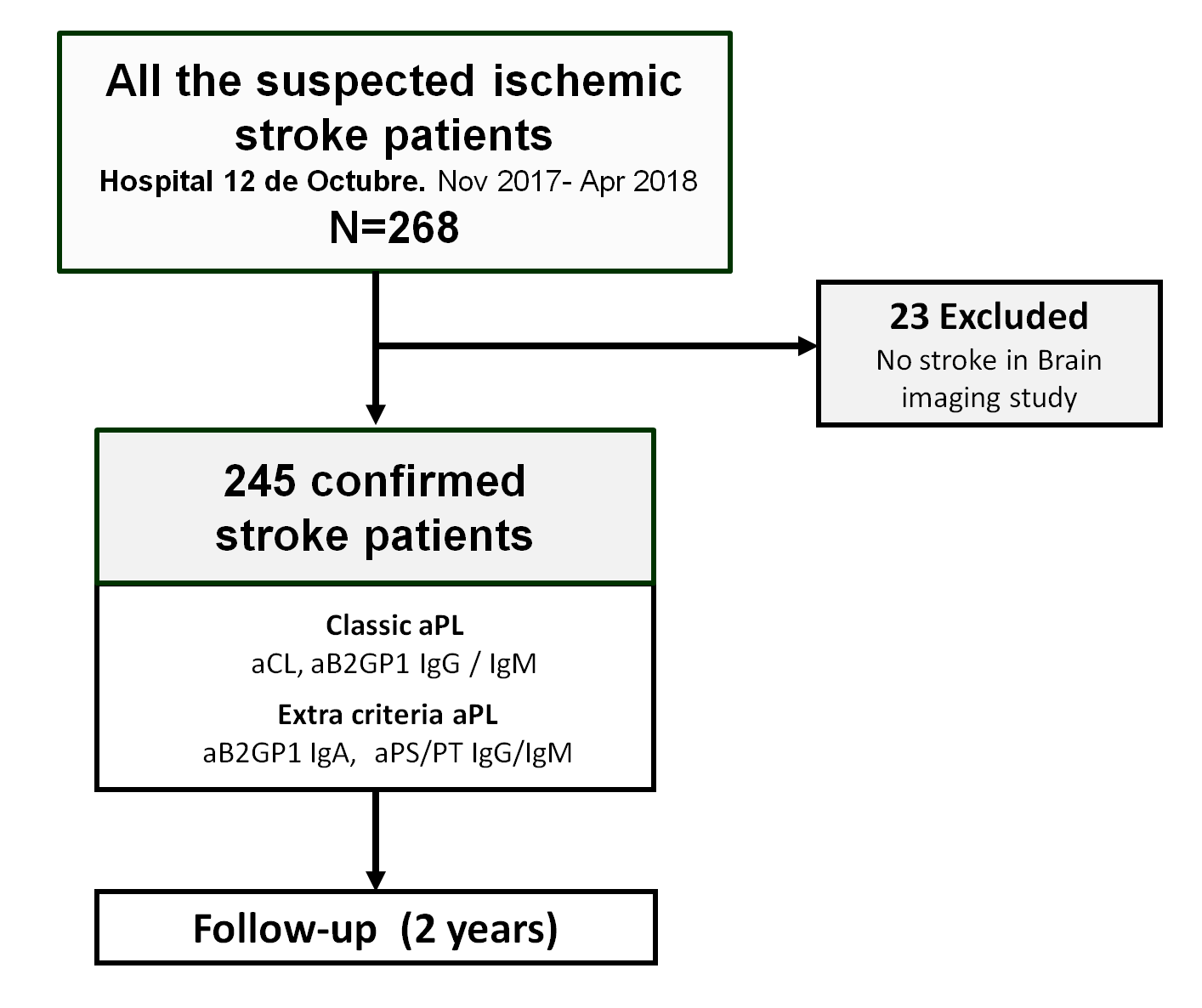

Supplement: Supplementary file 3 [file Image_1.TIF]

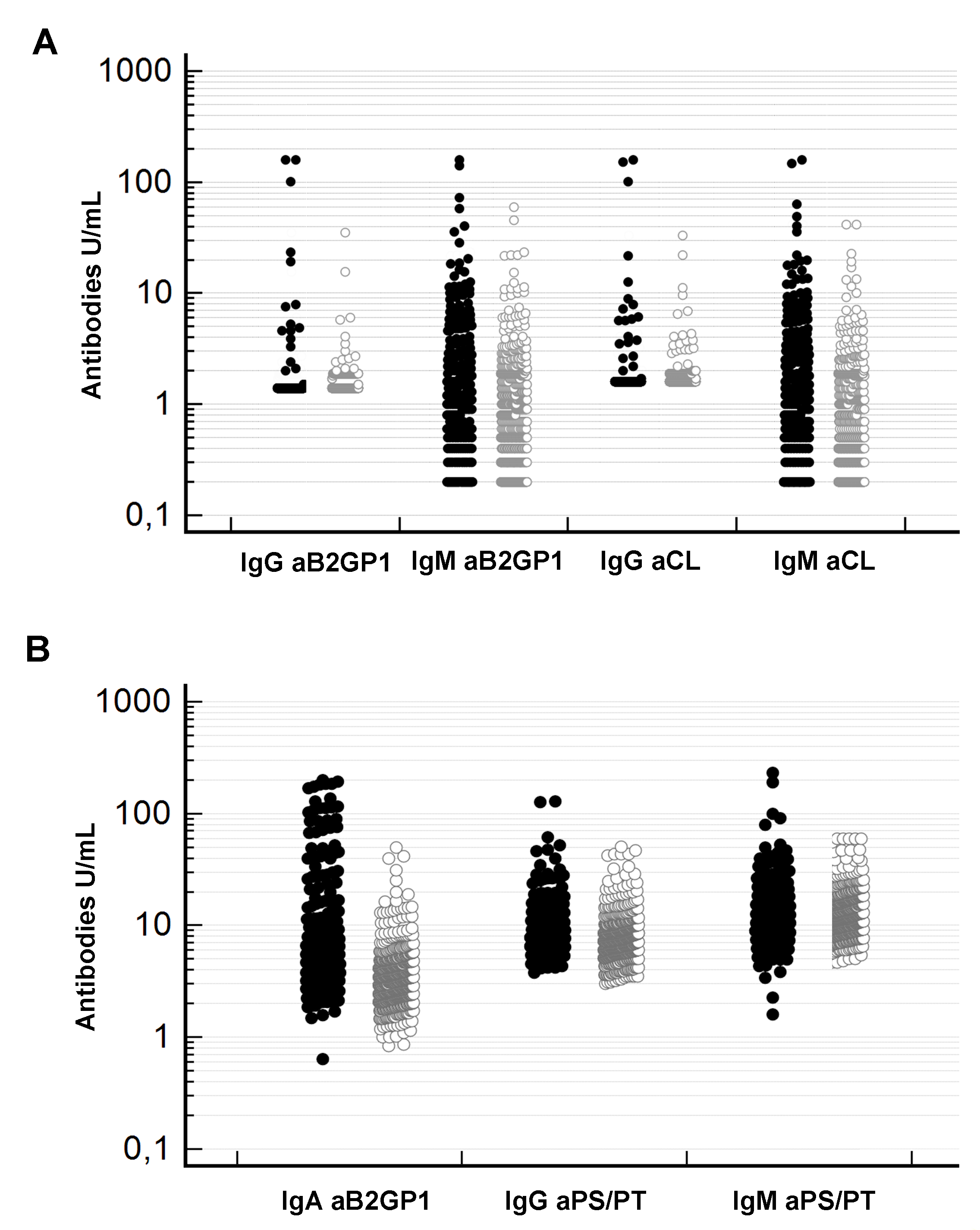

Supplement: Supplementary file 4 [file Image_2.TIF]
